# Honeycomb Sternum Mimicking Manubriosternal Dislocation in a Pediatric Trauma Patient

**DOI:** 10.7759/cureus.85137

**Published:** 2025-05-31

**Authors:** Noora AlSuwaidi, Sania Shahid, Saba Fatima, Amna Mohammed

**Affiliations:** 1 Pediatric Emergency Medicine, Al Jalila Children's Speciality Hospital, Dubai, ARE; 2 Pediatric Emergency Medicine, Dubai Academic Health Corporation, Dubai, ARE; 3 Emergency, Al Jalila Children's Speciality Hospital, Dubai, ARE

**Keywords:** blunt injury, chest injury, pediatric, sternum, trauma

## Abstract

This case report describes a four-year-old girl who presented to the emergency department after falling from a height. The initial assessment, including a chest X-ray, raised concern for a manubriosternal joint dislocation. To further evaluate the suspected sternal injury, blood tests and a CT chest were performed. These investigations revealed no signs of acute trauma but instead identified a normal anatomical variant, non-fused lateral ossification centers of the sternum, commonly referred to as “honeycomb sternum.” This case underscores the importance of recognizing developmental variants in pediatric patients to prevent misdiagnosis, unnecessary imaging, and avoidable referrals.

## Introduction

Sternal injuries in pediatric trauma are uncommon due to the pliability of the chest wall in young children [1]. When such injuries are suspected, clinicians must differentiate between true pathological findings and normal developmental variants. One such variant is the rare “honeycomb sternum,” characterized by non-fused ossification centers that can mimic fractures or dislocations on imaging [1,2].

The sternum develops from multiple ossification centers that appear in a segmental fashion during infancy and early childhood. Fusion of these centers typically occurs gradually over the first few years of life, although the timing can vary significantly among individuals [1,3]. Misinterpretation of these normal developmental features as traumatic injury may lead to unnecessary diagnostic testing or invasive interventions [3,4].

This case report aims to raise awareness of honeycomb sternum as a benign anatomical variant, particularly in the context of pediatric trauma, to prevent misdiagnosis and reduce avoidable investigations or referrals.

## Case presentation

A four-year-old girl presented to our emergency department after falling from a height of approximately two meters. During the fall, she initially struck the back of her head before flipping and landing on her chest. On initial assessment, the child experienced two episodes of vomiting and appeared lethargic, though she remained fully oriented to time, place, and person. Her Glasgow Coma Scale (GCS) score was 15/15. There were no reported episodes of abnormal movements, and she did not lose consciousness.

Prior to arriving at our facility, the patient had been evaluated at another healthcare center. A CT scan of the brain performed there revealed no acute intracranial pathology. A chest X-ray done at the outside facility reportedly showed an irregular sternal contour, which raised concern for a possible manubriosternal joint injury and led to her referral for further evaluation and cardiothoracic consultation. Unfortunately, the original chest X-ray images were not available for review at our center.

Upon arrival, the patient was sedated but easily arousable. She maintained a GCS of 15/15 throughout observation. Her vital signs were stable, and a thorough systemic physical examination was unremarkable. Initial laboratory investigations, including cardiac biomarkers (troponin I and T), coagulation profile, complete blood count, renal and liver function tests, and metabolic panel, were all within normal limits (Table 1).

**Table 1:** Blood test results INR: international normalized ratio, APTT: activated partial thromboplastin time, WBC: white blood cell, HCO3: bicarbonate, SGOT: serum glutamic oxaloacetic transaminase, AST: aspartate aminotransferase, SGPT: serum glutamic pyruvic transaminase, ALT: alanine aminotransferase

Lab	Value	Reference
Troponin I	<0.010	0.00-0.03 ng/ml
Troponin T	6	<11 ng/L
Prothrombin time	13.1	12.1-14.5 secs
INR	0.95	0.92-1.14
APTT	32.4	33.6-43.8 secs
WBC count	12.5	5.0-15.0 10^3/uL
Hemoglobin blood	12.4	11.1-14.1 g/dL
Platelet count	295	200-490 10^3/uL
Sodium	140	135-142 mmol/L
Potassium	4.4	3.9-5.4 mmol/L
Chloride	106	101-111 mmol/L
HCO3	23.3	14-24 mmol/L
Creatinine	0.25	0.3-0.5 mg/dL
Urea	29	12-40 mg/dL
Calcium	9.9	9.3-10.6 mg/dL
Glucose, random	87	72-112 mg/dL
SGOT (AST)	36	0-41 U/L
SGPT (ALT)	18	0-19 U/L
Alkaline phosphatase	252	142-335 U/L
Total protein	6.6	5.9-7.3 g/dL
Bilirubin, total	0.15	0-0.3 mg/dL
Albumin	4.2	4.0-4.9 g/dL

**Figure 1:**
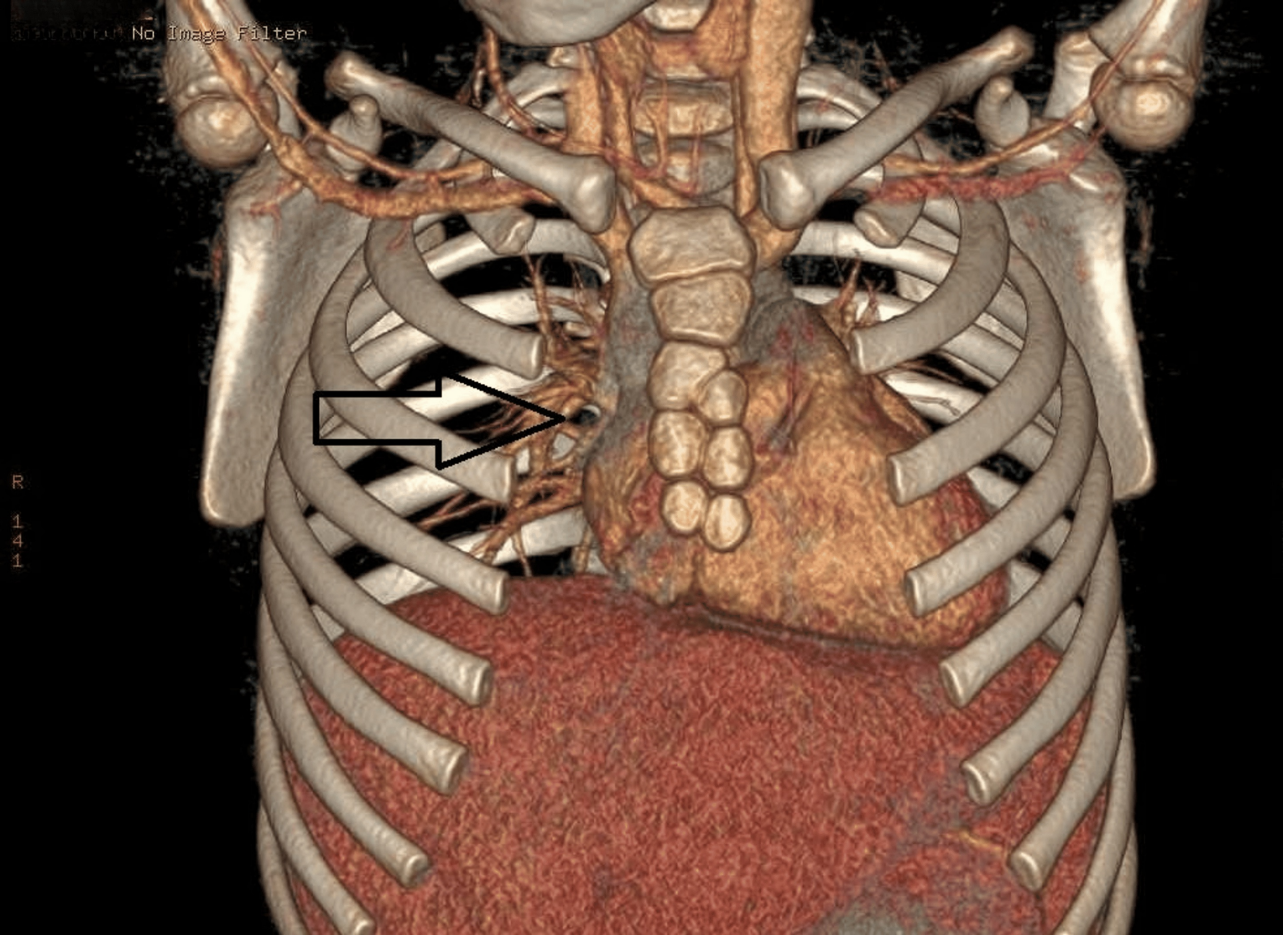
CT 3D deconstruction image showing the honeycomb appearance of the sternum CT: computed tomography, 3D: three dimensional

**Figure 2:**
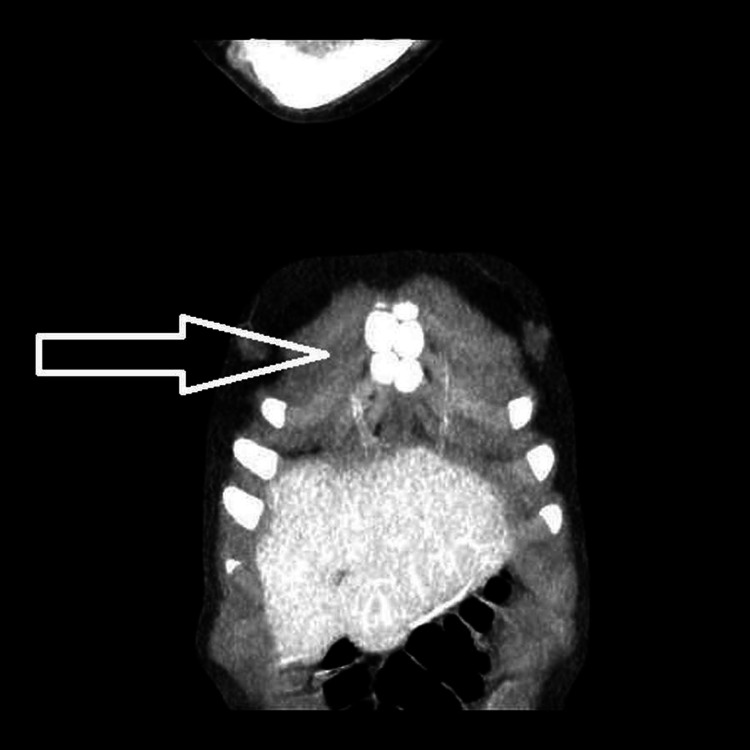
Coronal CT view demonstrating non-fused lateral ossification centers in the sternum CT: computed tomography

**Figure 3:**
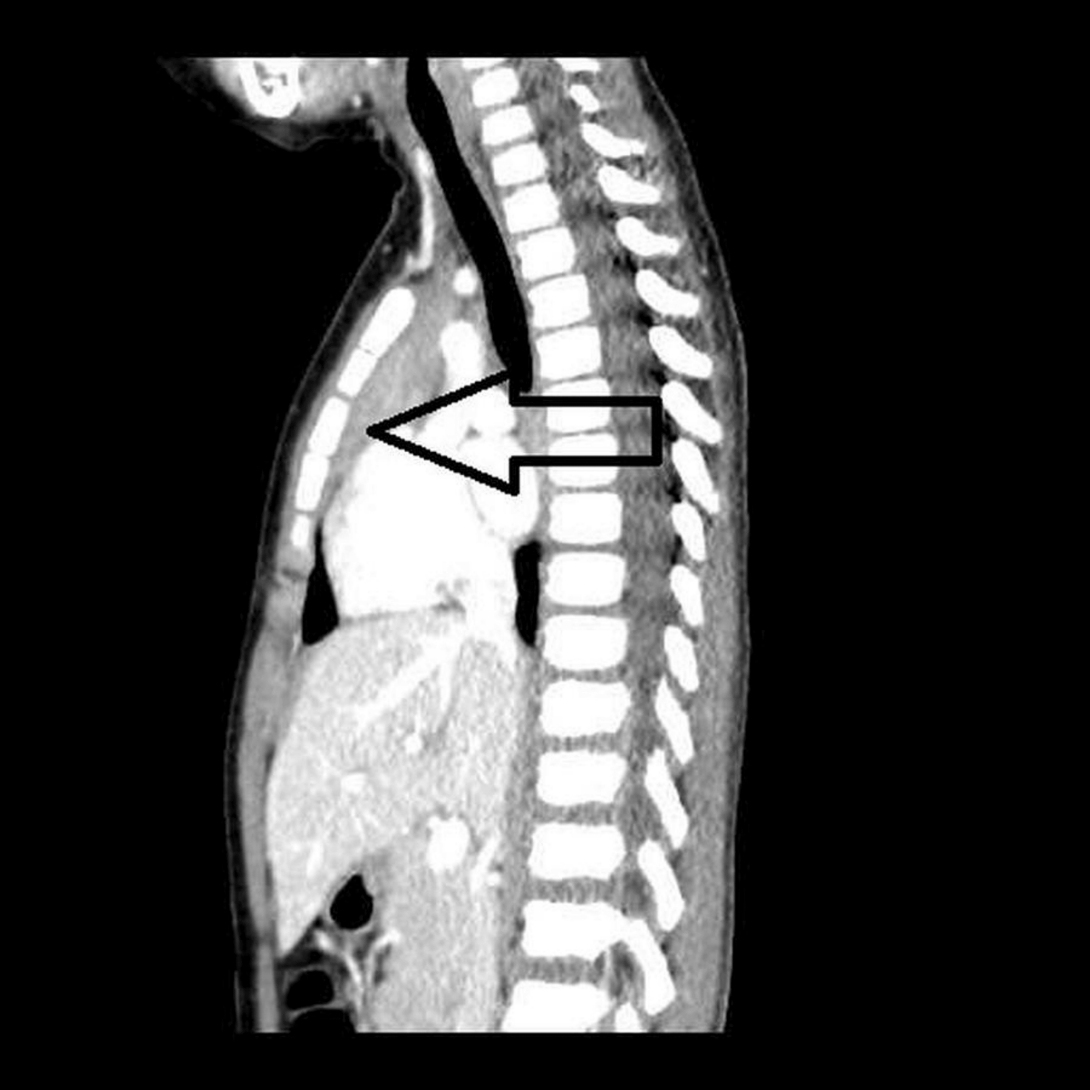
Sagittal chest CT showing absence of dislocation and normal mediastinal anatomy CT: computed tomography

To further assess the suspected sternal injury, a chest CT scan was performed (Figures 1-3). The CT revealed a normal tracheobronchial tree and mediastinal structures, with no evidence of pneumothorax, effusion, or fracture. Notably, there was no manubriosternal dislocation. Instead, the CT demonstrated non-fused lateral ossification centers within the sternum, producing a multi-lucent, trabeculated pattern.

A diagnosis of honeycomb sternum, a benign developmental variant, was made. Cardiothoracic consultation confirmed that no intervention was necessary. The child was discharged in stable condition after a short observation period with outpatient follow-up. She remained asymptomatic on follow-up visits.

## Discussion

Sternal injuries in the pediatric population are rare, largely due to the compliant nature of the thoracic cage. Children’s ribs and costal cartilages absorb energy more effectively than adults’, reducing the risk of bony trauma even in significant impacts [1]. When trauma does result in apparent skeletal findings, it is essential to differentiate between true injury and anatomical variations, particularly in high-pressure emergency settings.

One of the most commonly misinterpreted findings on pediatric chest imaging is the presence of unfused ossification centers within the sternum. These centers may remain distinct into adolescence and can vary widely between individuals [1,4]. In younger children, especially under the age of five, it is not uncommon to visualize gaps or “honeycomb” patterns in the sternal body on radiographs or CT. Without prior knowledge of these normal variants, clinicians and radiologists may raise concerns for sternal fractures or joint dislocations [1].

In our case, the initial concern was a manubriosternal dislocation. While most dislocations occur in the context of motor vehicle collisions or direct anterior thoracic impacts, they have occasionally been reported in falls. However, these injuries are almost always accompanied by chest wall tenderness, deformity, or cardiorespiratory compromise, none of which were observed in our patient [3].

Despite the absence of clinical signs of chest trauma, the initial radiological interpretation led to a cardiothoracic surgery referral. This highlights a recurrent issue in pediatric trauma care: overcalling injuries due to unfamiliarity with pediatric developmental anatomy. Several studies, including that by Winant et al., emphasize the importance of pediatric-specific imaging interpretation, as misdiagnoses often stem from adult-trained radiologists unfamiliar with normal variant appearances in children [1].

Moreover, ossification of the sternum follows a well-established but variable timeline. The manubrium and xiphoid process ossify from separate centers and fuse gradually during late childhood. The body of the sternum (mesosternum) forms from four to six sternebrae, which may remain partially unfused well into adolescence [4]. These ossification centers appear as discrete lucencies or irregularities on imaging and can mimic trauma-induced pathology, such as fractures, clefts, or even infections if calcifications are involved [4].

In our patient, the lateral non-fused ossification centers created a honeycomb-like appearance on CT. While this pattern is benign, other developmental variants, such as sternal clefts or bifid sternum, can also mimic traumatic findings and should be considered in the differential diagnosis [1].

The term “honeycomb sternum” is not widely adopted in formal literature. However, it is used colloquially to describe the radiographic appearance of multiple unfused sternal segments that resemble a lacy or trabeculated pattern. Radiologists and emergency physicians must correlate these findings with clinical presentation to avoid diagnostic errors [1].

This case also highlights the importance of obtaining second opinions and repeating imaging. In facilities where pediatric radiology expertise is limited, initial interpretations may be cautious or overly conservative. When clinical signs are inconsistent with imaging results, further review or consultation with subspecialists can help prevent overtreatment. Had our patient undergone unnecessary intervention based on the initial scan, she may have faced risks from anesthesia, radiation exposure, prolonged hospitalization, or even unwarranted surgery, all of which carry significant morbidity in pediatric care.

Pearson et al. point out that overtriage in pediatric thoracic trauma is a recognized issue, often stemming from imaging artifacts or developmental variations rather than true pathology [3]. In practice, this can lead to psychological stress for families, an economic burden on the healthcare system, and an erosion of patient trust [3]. Therefore, enhancing educational efforts for general radiologists and emergency physicians regarding normal pediatric anatomical variants could mitigate such outcomes.

Ultimately, this case highlights the importance of integrating radiological findings into the comprehensive clinical context. The child in our report had no respiratory distress, chest pain, or instability. Her blood work was normal, and systemic examination revealed no signs of trauma. These observations should have prompted caution before escalating care based solely on imaging. Clinicians must remain vigilant but also reflective, considering the developmental context of their patients before reaching a diagnosis.

## Conclusions

Misinterpreting the honeycomb sternum can lead to unnecessary referrals, anxiety, and interventions. The honeycomb sternum is a normal developmental variant in children. It represents non-fused ossification centers; in our case, it was misinterpreted as a traumatic injury from a fall from a height. Being able to identify this variant, in conjunction with various clinical assessments, is crucial for accurately and efficiently managing pediatric trauma.
